# Gluu Essentials Digital Skills Training for Middle-Aged and Older Adults That Makes Skills Stick: Results of a Pre-Post Intervention Study

**DOI:** 10.2196/50345

**Published:** 2023-11-10

**Authors:** Cherisse L Seaton, Kathy L Rush, Eric Ping Hung Li, Mohammad Khalad Hasan, Linda Fawcus

**Affiliations:** 1 School of Nursing University of British Columbia Kelowna, BC Canada; 2 Faculty of Management University of British Columbia Kelowna, BC Canada; 3 Department of Computer Science, Mathematics, Physics and Statistics University of British Columbia Kelowna, BC Canada; 4 Gluu Society Vancouver, BC Canada

**Keywords:** digital literacy, digital skills, older adults, mobile device proficiency, online, mobile phone

## Abstract

**Background:**

A number of real-world digital literacy training programs exist to support engagement with mobile devices, but these have been understudied.

**Objective:**

The purpose of this study was to examine the effectiveness and program acceptability of a digital skills training program among middle-aged and older adults (aged ≥50 years) and to gather participants’ recommendations for lifelong digital skills promotion.

**Methods:**

The Gluu Essentials digital skills training program includes learning resources to support tablet use. Through pre-post surveys, this study assessed mobile device proficiency, confidence in going online and in avoiding frauds and scams, the frequency of engaging in online activities, program engagement, acceptability, and suggestions for continued support.

**Results:**

A total of 270 middle-aged and older adults completed baseline surveys. Of these 270 participants, 145 (53.7%) completed follow-up surveys. Our findings indicate that mobile device proficiency increased (*P*<.001), whereas confidence was unchanged. Participants also reported going online more frequently to shop (*P*=.01) and access government services (*P*=.02) at follow-up. Program engagement varied considerably, but program acceptability was high. Participants’ recommendations included the need for providing ongoing programs for support and training because technology constantly changes, reducing costs for technology and internet access, and keeping learning resources simple and easy to access.

**Conclusions:**

The Gluu Essentials digital skills training program increased mobile device proficiency and frequency of web-based activities (shopping and accessing government services) among middle-aged and older adults.

## Introduction

### Background

In Canada and in many nations, there has been an increase in technology reliance, with many aspects of life now optionally or necessarily being navigated in digital terms; for example, online banking has become the norm in managing financial matters, and virtual health care options are becoming increasingly more available. We use the term *online* to refer to connecting to the internet for activities such as accessing websites, online services, and email. Although the internet can provide access to important financial, health, and social resources and information [1], the aging population has created a universal challenge to the ongoing digitalization of key services because older adults use the internet at lower rates than younger adults—a phenomenon known as the “grey digital divide” [2]. In 2022, the World Health Organization projected that 17% of the world’s population will be aged ≥60 years in 2030, increasing to 22% by 2050 (compared with 12% in 2015) [3]. According to the Pew Research Center, nearly 100% of young adults reported using the internet in 2022 in nearly every country surveyed, whereas use ranged from 57% to 93% among older adults; still, this use was much higher than in 2002, indicating that the number of older adults engaging with technology has been steadily increasing [4]. To meaningfully participate in society, older adults increasingly need digital literacy [5], or the skills to locate, understand, generate, organize, and evaluate information using digital technology [6].

Mobile and smartphone devices afford greater access to connectivity even in rural areas [7], yet one area where a greater gray digital divide has been reported is in mobile device uptake. Although nearly all young adults in the 18 countries surveyed reported smartphone ownership in 2022, the rates of ownership among adults aged ≥50 years in some countries were as low as 55% in 2022 [4]. Previous research showed that mobile devices offer advantages in terms of supporting independence over a PC (a computer in a fixed location) for communication, transportation, navigation, entertainment, and health [8]. Indeed, advanced personalization and user-centric apps have been developed for mobile devices over the recent past; for example, mobile health apps designed to remind users of upcoming appointments or medicine intake are much more useful if implemented on a mobile device that is carried with the user throughout the day [9].

However, extant literature reported that older adults face numerous barriers to digital technology adoption, such as a lack of confidence [10]; negative or ambivalent attitudes toward technology, including privacy concerns [11,12]; and the need for ongoing support from others [13,14]. To address these challenges, several programs have been introduced to promote digital literacy among middle-aged and older adults [15].

To date, the majority of digital literacy programs have been focused on increasing technology acceptance, adoption, use, or interest, with some success [16-21]. As technology has the potential to help older adults stay connected to friends and family, many technology-based interventions have been focused on reducing loneliness, but these have had mixed results, often attributed to failure to target socially isolated older adults [22-25]. Studies have reported that social support to set up new devices plays an important role in initiating and motivating tablet use among older adults [18] and that consideration of older adults’ needs, preferences, and lived experience is essential to success [17,19]. Indeed, a recent systematic review of instructional strategies to promote the learning of digital technology among older adults reported that collaborative, personalized, relevant, and experience-based learning strategies accompanied by repetition and effective teaching aids (such as print resources) were themes present throughout many of the 17 studies reviewed [26]. However, previous studies have reported primarily qualitative results [21] or were conducted in specific settings (eg, Castilla et al [20] examined training on Butler 2.0, a system application designed to increase social support) or in highly controlled settings (eg, the randomized controlled trial conducted by Arthanat [16]), with few real-world assessments of training implementation.

Indeed, in Canada, a number of digital literacy training programs exist, yet often these have been understudied. More research is needed on the impact of digital skills training on middle-aged and older adults’ digital engagement. Gluu Society is a Canadian nonprofit organization that provides free digital skills training to middle-aged and older Canadians [27]. Gluu is not an acronym; rather, it is a name selected to convey ensuring that digital skills *stick* and are not forgotten. A report on a national digital literacy program that incorporated the Gluu Essentials courses (ie, foundational training on tablet use) outlined a number of best practices for program delivery, including community-based and flexible program delivery [28]; however, as in the case of many existing digital literacy training programs, there has been no systematic evaluation of the impact of the Gluu Essentials program, particularly on middle-aged and older adults’ digital skills acquisition related to mobile device use. Measuring mobile device proficiency is 1 way to gauge the effectiveness of training [9]; however, few previous studies have focused on mobile device proficiency in particular [29].

### Objectives

The primary objective of this study was to examine the effectiveness of the Gluu Essentials training program for improving participants’ mobile device proficiency. The secondary objective was to explore the impact of the training on the frequency of participants’ engagement in online activities (eg, shopping, banking, and accessing government services); their confidence; and their satisfaction with, and acceptability of, the program.

## Methods

### Study Design

This study used a pre-post cross-sectional survey design in which data were collected from middle-aged and older adults in rural and urban communities in Canada before and after participation in the digital skills training program.

### Digital Skills Training Program

The digital skills training program was developed by Gluu Society and delivered in partnership with local community organizations and volunteer coaches. Gluu’s target audience consisted of adults aged ≥50 years. The Gluu Essentials resources included (1) printed student workbooks; (2) support for coach volunteers: training, lesson plans, and teaching resources; and (3) online support for participants and volunteers.

**Figure 1:**
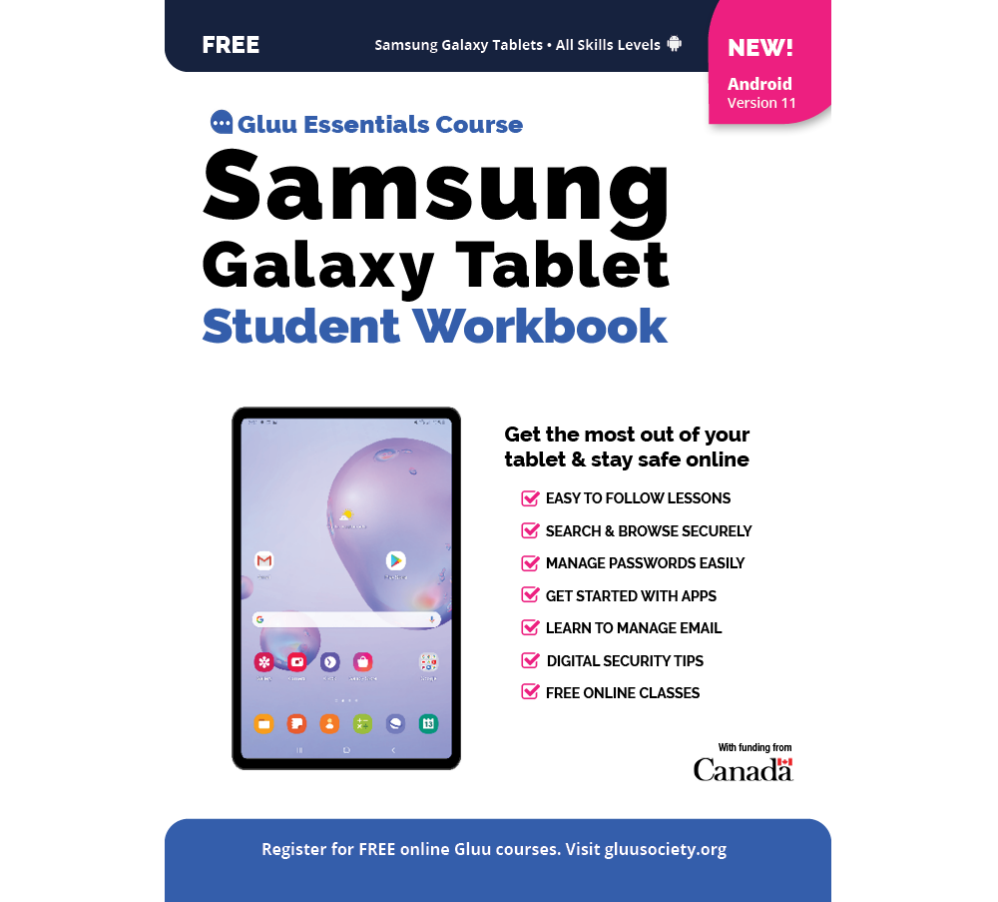
Cover of the Gluu Essentials Samsung Galaxy Tablet Student Workbook.

**Figure 2:**
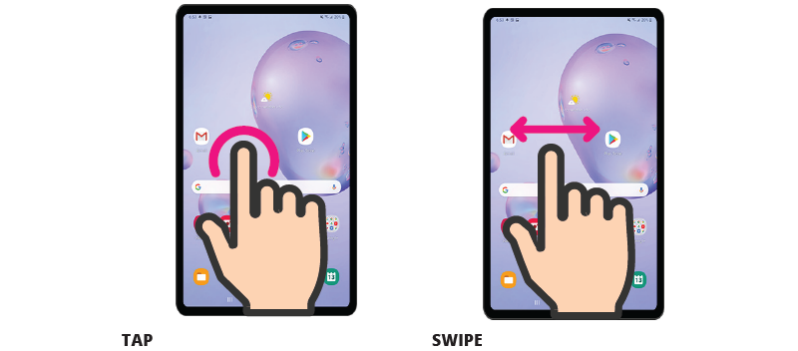
Examples of images depicting basic touch screen gestures.

The Gluu Essentials courses included learning resources to help middle-aged and older adults use Apple iPads or Samsung Galaxy tablets. Courses were centered around these devices because they are top sellers; have user-friendly interfaces; and their manufacturers emphasize security, ensuring that users have a safe digital experience. The printed workbooks included a 96-page Gluu Essentials iPad Student workbook and a 96-page Gluu Essentials Samsung Galaxy Tablet Student workbook (Figure 1). Within the workbooks, user-friendly images were used throughout to display touch screen actions (refer to the example gestures presented in Figure 2). Twelve lessons were included in the Gluu Essentials training program, beginning with (1) the basic features of the tablet (eg, overview of where everything is and powering the tablet on and off) followed by (2) an introduction to the touch screen and gestures, (3) settings and tablet care, (4) the camera app, (5) email as well as Gmail or email app, (6) managing contacts, (7) find what is needed online, (8) how to download apps, (9) using the calendar app, (10) digital security basics, (11) Facebook basics, and (12) Zoom basics. Although Gluu recommended 3 months of weekly classes of no more than an hour to cover each topic (ideally with at least 70% attendance), community organizations and volunteer coaches were free to deliver the programming in the way that worked best for their learners and staff availability. Thus, program duration varied across participants according to the self-paced nature of the delivery, and we were unable to track programming delivered by local community organizations in this real-world assessment. The Gluu Essentials training was provided free of charge; however, the devices were not provided by Gluu.

### Sample Recruitment and Procedures

Organizations in 23 communities across Canada (n=14, 61% in British Columbia) were interested in partnering with Gluu to deliver digital skills training. A total of 820 paper surveys (in batches of 10-60) were sent to the community organizations along with the printed Gluu Essentials workbooks. The paper surveys included postage-paid return envelopes addressed to the University of British Columbia (Okanagan Campus) research team. Approximately half of the organizations (10/23, 43%) fully engaged with the Gluu Essentials program (ie, recruited a combined total of 36 volunteers to complete Gluu’s volunteer training program and join a Slack workspace—a digital platform for coach-to-coach communication managed by the Gluu Support Crew Leader); of the 10 organizations that stayed connected to Gluu one-third (3/10, 30%) provided lessons in person (eg, small groups), and two-thirds (7/10, 70%) had volunteers who provided support to learners from a distance (eg, telephone support). At least 1 other organization that we were aware of delivered the workbooks and training in-person and without Gluu support, but we were unable to track how programming was delivered by the remaining organizations that did not stay connected to Gluu. The online survey was also advertised on Gluu’s Facebook page in fall 2022, emailed to Gluu’s mailing list, and sent through a provincial community response network with >85 rural community affiliates. Online survey participants were individually mailed their preference of Gluu Essentials iPad or Samsung Galaxy Tablet Student workbooks. In terms of inclusion and exclusion criteria, although the recruitment advertisements were targeted at middle-aged and older adults, they only specified that participants must be Canadian residents; however, only participants aged ≥50 years were recruited for follow-up and included in the study analysis.

On the baseline survey, participants were asked to check whether they were willing to be contacted for a follow-up survey. Those who selected *yes* were sent up to 5 follow-up invitations by email, and those who provided mailing addresses were mailed paper follow-up surveys with postage-paid return envelopes. To promote participation, three CAD $100 (US $75) draw prize incentives were advertised after the completion of baseline data collection and again after the completion of the follow-up survey.

### Ethical Considerations

All participants provided informed consent before completing the survey. This study was reviewed by, and received ethics approval from, the University of British Columbia’s behavioral research ethics board (H21-02116).

### Measures

Sociodemographics, participant characteristics, and postal codes were gathered at baseline. Self-report measures of mobile device proficiency, confidence, and the frequency of engaging in online activities were collected at both baseline and follow-up. Finally, several questions exploring program engagement, acceptability, and suggestions were completed at follow-up only. Tables 1 and 2 present a detailed description of all survey measures.

**Table 1:** Summary of study measures collected at baseline only and those collected at both baseline and follow-up.

Time point and name		Description	Response options and score range	Score calculation	Psychometric and interpretation information
**Measures collected at baseline only**					
	Sociodemographic characteristics	Age, sex, ethnicity, education, and devices used (ie, desktop computer, smartphone, or tablet)	N/A^a^	N/A	N/A
	Community and remoteness	Participants provided their postal code, and by using the Canadian *Find a postal code* tool [30], community or city name and province were determined; based on the census subdivision of the community, a score was assigned from Statistics Canada’s Index of Remoteness (RI^b^ scores are based on population size and cost to travel to nearest population center [31])	Possible scores range from 0 to 1; the method developed by Subedi et al [32] was used to classify each community’s RI score into 1 of 5 categories: easily accessible (<0.1500), accessible (0.1500-0.2888), less accessible (0.2889-0.3898), remote (0.3899-0.5532), or very remote (>0.5532)	*Easily accessible* and *accessible* were further collapsed as *urban*, and *less accessible*, *remote*, and *very remote* were categorized as *rural*	Scores closer to 1 indicate greater remoteness
**Measures collected at both baseline and follow-up**					
	Mobile device proficiency	Participants responded to 18 items (eg, “Using a mobile device I can use the onscreen keyboard to type”) from the MDPQ^c^ [9]; we included items to measure 7 MDPQ subscales: mobile device basics (2 items), communication (3 items), internet (3 items), calendar (2 items), entertainment (2 items), privacy (2 items), and troubleshooting or software management (4 items)	Response options range from 1 (*never tried*) to 5 (*very easily*)	The mean of all 18 items was computed as an overall measure of digital literacy, and the mean of responses to the items making up each subscale were also computed	Higher scores represent greater literacy; the MDPQ has demonstrated internal reliability as well as convergent and divergent validity in previous research [9,33]; in this study, the Cronbach α value for the overall MDPQ scores was high (.96)
	Confidence in going online and confidence in online security	On the basis of the 1977 study by Bandura [34], single investigator-developed items asked participants about their confidence in going online (“Over the next 6 months, how confident are you about going online [eg, to use websites, online services, etc.]?”) and about online security (“Over the next 6 months, how confident are you about identifying online frauds and scams?”)	Both items were rated on a Likert scale ranging from 1 (*not at all confident*) to 5 (*extremely confident*)	N/A	Single-item measures of self-efficacy have demonstrated moderate correlations with multi-item scales as well as correspondence with measured outcomes [35]
	Frequency of online activities	Questions were adapted from the MTUAS^d^ emailing and internet subscales (Rosen et al [36]) to assess participants’ frequency in going online for 7 different activities (sending and receiving emails, online shopping, online banking, accessing government services, searching for information, accessing COVID-19–related information, and accessing emergency preparedness information or alerts)	Ordinal response choices included 1 (*never*), 2 (*once a year*), 3 (*several times a year*), 4 (*once a month*), 5 (*several times a month*), 6 (*once a week*), 7 (*several times a week*), 8 (*once a day*), and 9 (*several times a day*)	Binary variables were also created by grouping participants who responded with *never* versus those who responded with any frequency above *never*	N/A

^a^N/A: not applicable.

^b^RI: remoteness index.

^c^MDPQ: Mobile Device Proficiency Questionnaire.

^d^MTUAS: Media and Technology Usage and Attitudes Scale.

**Table 2:** Summary of study measures collected at follow-up only.

Name	Description	Response options and score range	Score calculation	Psychometric and interpretation information
Program engagement	Participants were asked to indicate the portion they had completed of each of the 12 lessons included in the Essentials training; they were also asked the following questions: “To what extent did you use the printed Gluu Workbook?” “How did you access a Digital Coach?” “Did you view materials/posts on Gluu social media via Facebook or Instagram?” “Are you still using the Gluu digital skills resources?”	Response options for each of the 12 lessons ranged from 0 (*did not start*) to 4 (*completed*), or participants could indicate *unsure*; workbook use response options included 0 (*did not use*), 1 (*used once a month or less*), 2 (*used several times a month*), 3 (*used weekly*), and 4 (*used daily*); digital coach response options included 0 (*I did not*) and 1 (*by telephone or in-person classes*); those who accessed a coach by telephone or in person were asked to specify how many times they did so; social media and continued use of Gluu resources response options were 0 (*no*) or 1 (*yes*)	Participants were given a score of 1 (*yes*) if they indicated starting at least 1 of the Gluu Essentials lessons and a score of 0 (*no*) if they selected *did not start* in response to all 12 lessons; participants were given dichotomous scores based on engagement (*yes*) or not (*no*) with each of the program components	N/A^a^
Program acceptability	Participants were asked 4 questions (“Overall I was satisfied with the Gluu digital skills training,” “I learned new ways to use my device through the Gluu digital skills training,” “I learned new information about online security through the Gluu digital skills training,” and “After this training, I am more likely to use technology to support my aging plan [eg, aging in place]”); finally, the 10-item SUS^b^ [37] was adapted to refer to the Gluu resources (eg, “The Gluu resources were easy to use”)	Response options for acceptability questions ranged from 1 (*strongly disagree*) to 5 (*strongly agree*); for the SUS, response options ranged from 0 (*disagree*) to 4 (*agree*)	Ratings of *agree* and *strongly agree* were combined to denote acceptability; for the SUS, 5 negatively worded questions were reverse scored, and responses to items were summed and normed on a scale ranging from 1 to 100, with higher scores reflecting greater perceptions of usability	Scores of >68 are considered above average in terms of usability [38]
Open-ended suggestions	Participants were asked, “What would support you to be on a lifelong learning digital skills journey?”	N/A	Open-ended responses were reviewed; informative quotes were extracted using inductive thematic analysis	N/A

^a^N/A: not applicable.

^b^SUS: System Usability Scale.

### Data Processing and Statistical Analysis

Data were analyzed using SPSS for Windows (version 28.0; IBM Corp) [39]. The baseline characteristics of participants who completed the follow-up survey and those who did not were compared to determine whether the 2 subsets of participants differed on any variables at baseline. Fisher exact tests were used for categorical variables, independent 2-tailed *t* tests were used for continuous variables, and Mann-Whitney *U* tests were used for ordinal frequency of going online for different activities variables.

Separate paired samples 2-tailed *t* tests were used to compare baseline and follow-up Mobile Device Proficiency Questionnaire (MDPQ) and confidence variables. Wilcoxon signed rank comparisons were conducted to compare the baseline and follow-up frequency of online activities variables. In addition, separate linear mixed models for repeated designs were conducted following the intent-to-treat principle to compare baseline and follow-up MDPQ scores, as well as frequency and confidence variables to provide confirmation of the findings from the *t* tests and Wilcoxon signed rank comparisons. The models included a fixed effect for time and an unstructured covariance structure (which provided improved model fit over more basic compound symmetry models for all variables).

Furthermore, change in MDPQ scores was calculated by subtracting baseline scores from follow-up scores, with higher numbers representing greater increases in proficiency. Independent samples 2-tailed *t* tests were used to compare changes in MDPQ scores according to program engagement variables. All variables were inspected for normality and outliers. A *P* value of <.05 was considered statistically significant. Open-ended responses were analyzed by 2 research team members (CS and KR), and inductive thematic analysis was used to code and determine central themes.

## Results

### Survey Completion

**Figure 3:**
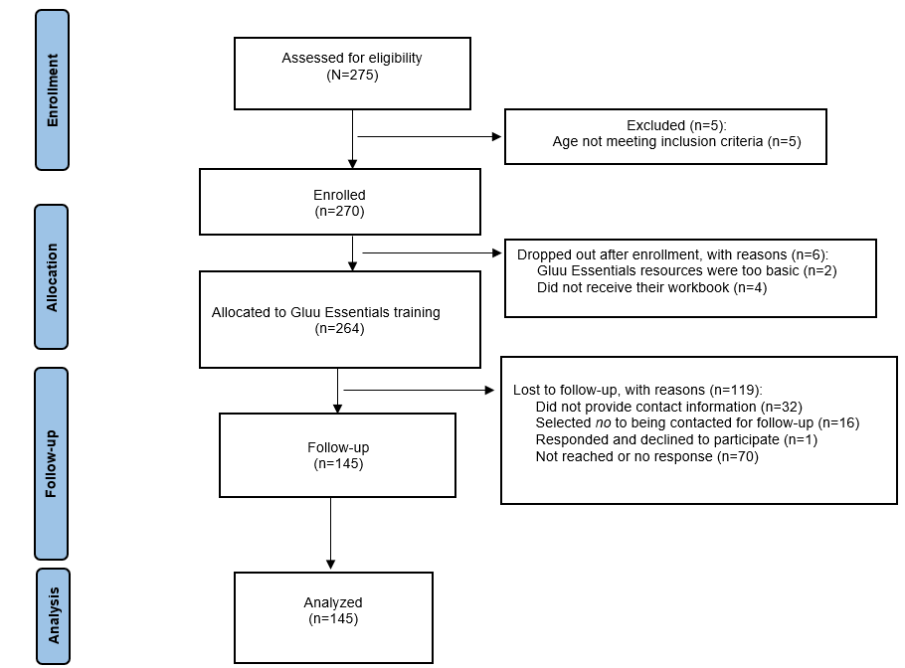
CONSORT (Consolidated Standards of Reporting Trials) flow diagram modified for nonrandomized trial design.

A total of 275 participants completed baseline surveys (n=8, 2.9% paper surveys in fall 2021; n=55, 20% paper surveys in spring or summer 2022; and n=61, 22.2% paper surveys and n=151, 54.9% online surveys in fall 2022). Of these 275 participants, 5 (1.8%) aged <50 years (aged 37, 39, 42, 45, and 47 years) were removed, and 6 (2.2%) subsequently dropped out, resulting in 264 (96%) enrolled in training at baseline. Of these 264 participants, 145 (54.9%) completed follow-up surveys (Figure 3). Participants completed follow-up surveys an average of 19.5 (SD 5.8) weeks after completing their baseline surveys.

### Sample Characteristics

The mean age of the participants was 72.93 (SD 6.73; range 51-93) years, and they were primarily female, White, and residing in the province of British Columbia (refer to Table 3 for participant characteristics at baseline, separated by whether or not follow-up surveys were completed). There were no differences between participants who completed the follow-up survey and those who did not in terms of age, ethnicity, education, or province; however, more of the female participants completed the follow-up survey than male participants, more of the urban participants completed the follow-up survey than rural participants, and more of the participants who completed online surveys at baseline completed the follow-up survey than those who completed paper surveys at baseline. More participants who used computers and tablets and used more apps completed the follow-up survey.

**Table 3:** Participant characteristics and mean scores on all variables at baseline (n=270).

Characteristics			All participants at baseline, n (%)		Follow-up survey completers (n=145), n (%)		Missing follow-up survey (n=125), n (%)		*P* value^a^	
**Age (years)**										.91
	50-64	27 (10)		12 (8.3)		15 (12.3)				
	Just answered “65+”	10 (3.7)		4 (2.8)		6 (4.9)				
	65-69	44 (16.3)		25 (17.2)		19 (15.6)				
	70-74	80 (29.6)		46 (31.7)		34 (27.9)				
	75-79	69 (25.6)		36 (24.8)		33 (27)				
	80-84	24 (8.9)		14 (9.7)		10 (8.2)				
	85-89	8 (3)		4 (2.8)		4 (3.2)				
	≥90	2 (0.7)		1 (0.7)		1 (0.8)				
**Sex**										.002
	Female	227 (84.1)		132 (91.7)		95 (77.2)				
	Male	40 (14.8)		12 (8.3)		28 (22.4)				
	Other	1 (0.4)		0 (0)		1 (0.8)				
	Prefer not to answer	1 (0.4)		1 (0.7)		0 (0.0)				
	Missing	1 (0.4)		0 (0)		2 (1.6)				
**Ethnicity**										.51
	Asian	7 (2.6)		5 (3.4)		2 (1.6)				
	Indigenous (First Nations or Métis)	19 (7)		7 (4.8)		12 (9.6)				
	Latin and South American	1 (0.4)		1 (0.7)		0 (0)				
	White	230 (85.2)		127 (87.6)		103 (82.4)				
	Other (eg, Canadian)	6 (2.2)		4 (2.8)		2 (1.6)				
	Prefer not to answer	3 (1.1)		1 (0.7)		2 (1.6)				
	Missing	4 (1.5)		0 (0)		4 (3.2)				
**Highest education level**										.31
	No high school diploma	14 (5.2)		4 (2.8)		10 (8)				
	High school diploma	44 (16.3)		24 (16.6)		20 16)				
	Trade or college diploma	44 (16.3)		22 (15.2)		22 (17.6)				
	Some college or university	45 (16.7)		27 (18.6)		18 (14.4)				
	University degree	113 (41.9)		64 (44.1)		49 (39.2)				
	Other	6 (2.2)		2 (1.4)		4 (3.2)				
	Prefer not to answer	2 (0.7)		2 (1.4)		0 (0)				
	Missing	2 (0.7)		0 (0)		2 (1.6)				
**Province**										.32
	British Columbia	233 (86.3)		126 (86.9)		107 (85.6)				
	Alberta	3 (1.1)		3 (2.1)		0 (0)				
	Manitoba	26 (9.6)		12 (8.3)		14 (11.2)				
	Saskatchewan	2 (0.7)		2 (1.4)		0 (0)				
	Ontario	3 (1.1)		1 (0.7)		2 (1.6)				
	New Brunswick	1 (0.4)		1 (0.7)		0 (0)				
	Missing	2 (0.7)		0 (0)		2 (1.6)				
**Remoteness index (rural or urban)**										.001
	Easily accessible	107 (39.6)		69 (47.6)		38 (30.4)				
	Accessible	32 (11.9)		20 (13.8)		12 (9.6)				
	Less accessible	56 (21.5)		24 (16.6)		32 (25.6)				
	Remote	58 (21.5)		28 (19.3)		30 (24)				
	Very remote	15 (5.6)		4 (2.8)		11 (8.8)				
	Missing	2 (0.7)		0 (0)		2 (1.6)				
**Survey format**										<.001
	Online	147 (54.4)		97 (66.9)		50 (40)				
	Paper	123 (45.6)		48 (33.1)		75 (60)				
“**Which device(s) do you use?”**										
	Computer	195 (72.2)		113 (77.9)		82 (65.6)		.02		
	Smartphone	213 (78.9)		118 (81.4)		95 (76)		.23		
	Tablet	208 (77)		121 (83.4)		87 (69.6)		.005		
	“I don’t use any device yet”	3 (1.1)		0 (0)		3 (2.4)		N/A^b^		
	Missing	2 (0.7)		2 (1.4)		0 (0)		N/A		

^a^*P* value based on Fisher exact tests (with missing data excluded). Comparisons for sex were dichotomized to male or female and for remoteness to rural or urban.

^b^N/A: not applicable.

Multimedia Appendix 1 presents a full breakdown of participant responses to all study variables at baseline separated by whether or not follow-up surveys were completed. Briefly, participants who completed the follow-up survey had higher values at baseline in overall mobile device proficiency, as well as higher values on the mobile device basics, internet, entertainment, privacy, and troubleshooting subscales, than those who did not complete the follow-up survey. Participants who completed the follow-up surveys also had higher confidence in going online as well as higher confidence in avoiding frauds and scams and were going online more frequently at baseline for email, accessing government services, information, and accessing COVID-19–related information than noncompleters.

### Pre-Post Differences in Mobile Device Proficiency, Confidence, and Frequency of Online Activities

Table 4 presents descriptive data for mobile device proficiency and confidence at baseline and follow-up along with results of the pre-post comparisons. Overall MDPQ scores improved from baseline to follow-up. Specifically, the largest improvements were seen in the mobile device basics, communication, internet, and troubleshooting subscales. On the basis of Cohen *d* values, the effects were small to approaching medium. Confidence in going online and in avoiding frauds and scams did not change significantly from baseline to follow-up.

The comparison of the groups (ie, Wilcoxon signed rank tests) shows that the frequency of going online for shopping (*P*=.01) and accessing government services (*P*=.02) increased, whereas the frequency of going online for email (*P*=.47), banking (*P*=.10), information (*P*=.96), and emergency services (*P*=.42) did not change significantly. The frequency of going online for COVID-19–related information significantly decreased (*P*=.01). Inspecting the distributions of responses to these ordinal scales showed that many of the participants who had selected *never* at baseline seemed to have instead selected some frequency above *never* at follow-up (except in the case of going online for COVID-19–related information). To aid the interpretation of these ordinal scales, Multimedia Appendix 2 displays the proportion of participants (n=145) who reported engaging in each of the online activities at any frequency above *never* at baseline and follow-up.

**Table 4:** Mobile device proficiency and confidence at baseline and at follow-up (n=145).

Variables			Baseline survey, mean (SD)		Follow-up survey, mean (SD)		*t* test^a^ (*df*)		*P* value		Cohen *d*
MDPQ^b^ total score			3.93 (0.91)		4.13 (0.79)		4.46 (143)		<.001		0.37
**MDPQ** **subscales**											
	Mobile device basics	4.32 (0.87)		4.50 (0.68)		3.36 (143)		<.001		0.28	
	Communication	4.32 (0.94)		4.45 (0.88)		2.17 (137)		.03		0.18	
	Internet	4.13 (0.99)		4.41 (0.79)		4.66 (137)		<.001		0.40	
	Calendar	3.58 (1.48)		3.64 (1.48)		0.73 (137)		.47		0.06	
	Entertainment	3.49 (1.33)		3.55 (1.26)		0.79 (136)		.43		0.07	
	Privacy	3.55 (1.10)		3.65 (1.03)		1.44 (137)		.15		0.12	
	Troubleshooting	3.91 (1.08)		4.16 (0.95)		4.14 (143)		<.001		0.35	
Confidence in going online			3.74 (0.91)		3.71 (0.88)		−0.45 (135)		.66		−0.04
Confidence in identifying frauds and scams			3.04 (0.73)		3.08 (0.77)		0.62 (137)		.54		0.05

^a^Separate paired samples 2-tailed *t* tests were used to compare baseline and follow-up scores among participants who completed surveys at both time points. Linear mixed models were also conducted following the intent-to-treat principle. The pattern of results was the same, although this was more pronounced in the linear mixed model analyses; therefore, the more conservative paired samples 2-tailed *t* test results are reported.

^b^MDPQ: Mobile Device Proficiency Questionnaire.

### Program Engagement and Change in Mobile Device Proficiency

Change in mobile device proficiency (ie, baseline MDPQ scores subtracted from follow-up scores), ranged from −0.94 to 2.78 (mean 0.20, SD 0.53). The negative numbers denote that, for some of the participants (43/144, 29.9%), the MDPQ scores actually decreased slightly from baseline to follow-up. Nevertheless, the MDPQ scores increased for the majority of the participants (101/144, 70.1%), with the greatest increase being 2.78 points on the 5-point scale. Change in mobile device proficiency also varied with some program engagement metrics. Table 5 presents changes in mobile device proficiency according to program engagement. At follow-up, the majority of the participants (105/134, 78.4%) who responded reported starting at least 1 of the Gluu Essentials lessons, and those who started or completed some lessons had a greater change in mobile device proficiency than those who did not start any lessons. Of the 138 participants who responded, 29 (21%) reported having a digital coach and reported taking between 0 and 15 (mean 6.41, SD 3.62) in-person, typically weekly, classes. Of the 143 participants who responded, 26 (18.2%) reported that they did not use the Gluu Essentials workbook, 53 (37.1%) used it once a month or less, 42 (29.4%) used it several times a month, 20 (14%) used it weekly, and 2 (1.4%) used it daily. Of the 141 participants who responded, 103 (73%) received the iPad workbook, 24 (17%) received the Samsung Galaxy Tablet workbook, 2 (1.4%) received both, 5 (3.5%) reported using the workbook for other devices (eg, *smartphone* or *laptop*), and 7 (5%) did not receive or use a workbook. Having a digital coach, using a printed workbook, and following Gluu on social media were all unrelated to change in mobile device proficiency; however, participants who reported still accessing Gluu resources had a greater change in mobile device proficiency than those no longer accessing these resources.

**Table 5:** Change in mobile device proficiency by program engagement (n=144).

Program engagement			Change in mobile device proficiency, mean (SD)		*t* test (*df*)			*P* value	
**Started or completed some lessons**						2.78 (132)			.007
	Yes (n=105)	0.24 (0.58)							
	No (n=29)	0.03 (0.28)							
**Had a digital coach**						1.23 (136)			.22
	Yes (n=29)	0.31 (0.61)							
	No (n=109)	0.17 (0.50)							
**Followed Gluu on social media**						0.07 (129)			.94
	Yes (n=54)	0.16 (0.50)							
	No (n=77)	0.15 (0.41)							
**Used printed workbook**						0.93 (140)			.36
	Yes (n=116)	0.22 (0.55)							
	No (n=26)	0.11 (0.36)							
**Still accessing Gluu Essentials resources**						2.17 (136)			.03
	Yes (n=94)	0.27 (0.55)							
	No (n=44)	0.07 (0.41)							

### Program Acceptability

Participant feedback on the Gluu Essentials training program was positive: 88% (82/93) reported being satisfied with Gluu Essentials digital skills training, 82% (75/92) reported learning new things about their device, 78% (73/93) reported learning new things about digital security, 78% (74/95) agreed or strongly agreed that the printed workbook was important, and 69% (60/87) stated that they were more likely to use technology to support their aging plan (eg, aging in place). System usability scores ranged from 25 to 90 (mean 72.76, SD 9.39), with 76 (79%) of the 96 participants who completed this scale assigning a score of >68 (ie, *above average*) to the Gluu Essentials program.

### Open-Ended Suggestions to Enable Lifelong Digital Skills Learning

Participants’ open-ended responses reflected 3 main themes: attitudes, program preferences, and support needs.

#### Theme 1: Attitudes

Many of the participants expressed positive attitudes toward digital skills training in general and the Gluu Essentials training specifically to keep up with changing technology and as part of their lifelong learning journey. A handful of participants were negative about such a digital skills journey—in some cases, even admitting agism—conveying that it was not for them and that “the technology changes all the time” and was “all too much” for them.

#### Theme 2: Program Preferences

Participants shared several sentiments about their program preferences and ways to enhance or supplement the program. Many enjoyed the availability of printed material, with some requesting the next level or step; some indicating ongoing programming preferences, such as “weekly newsletters” or “tips”; and some referring to the need for learning supports to be “simple,” “easy to access,” and “easy to follow,” as well as paced to learning needs, with 1 skill being practiced at a time.

#### Theme 3: Support Needs

Participants highlighted 2 principal support needs: human support and affordability support. Their predominant *human* support need was for one-on-one people support through coaching, accessing information, or calling a help desk if needed. Several participants mentioned that “reduced costs” for internet and new technology would support them. Textbox 1 displays several participant quotes corresponding to the emergent themes from the analysis of the open-ended suggestions.

**Responses to the open-ended question (“What would support you to be on a lifelong learning digital skills journey?”) gathering participant suggestions for supporting their lifelong digital skills learning journey (n=107).**

**Attitudes**

- “A continued program such as Gluu that I am able to access as I age, to keep pace with the constant changing technology that we elderly are facing.”
- “I feel very fortunate to have access to digital help through several sources in the community as well as through friends whose skills are more extensive than mine.”
- “I would only learn something if I can see a value in learning the task. I feel I am a lifelong learner. Always looking things up or reading about something new.”
- “I am convinced I need to keep on learning.”
- “The need to keep up with the technology.”
- “If that is even remotely possible. My days are numbered.”
- “I have no interest on being on such a digital journey.”
- “At my age I don’t have enough time. Would eventually surpass my learning skills.”

**Program preferences**

- “I need print material to refer to.”
- “I like having clear instructions like the workbook has.”
- “Your workbook was helpful. If there was another with more advanced learning that would be great.”
- “I would love another GLUU workbook at the next level up from basic.”
- “Weekly or bi-weekly newsletters, workshops, etc.... will there be any other online lessons?”
- “Perhaps a scheduled regular monthly or bimonthly webinar with updates and tips.”
- “Regular newsletter or emails with the tip of the week is one possibility.”
- “Easily accessible link that would take an individual through one aspect of technology each week with an opportunity to practice that one skill through the week. Too much information at once gets complicated.”

**Supports**

- “A person to help with my iPad. It’s difficult to look at the book without some help.”
- “An actual one-on-one REAL live coach at my side when needed. I really appreciate the support at my local library with one-on-one help.”
- “Talking with other people in the classes helps be prepared for different problems.”
- “More in-person workshops with Gluu. Also, access to a helpdesk would be most helpful.”
- “Access to a chat/support system.”
- “Ongoing person available for 1 to 1 learning, having money and access to keep up with new technology.”
- “To have enough money to afford newer devices.”
- “Cheaper costs for everything.”
- “A less exorbitant cost to internet service and cell service.”

## Discussion

### Principal Findings

The purpose of this study was to examine the effectiveness of the Gluu Essentials digital skills training program for improving participants’ mobile device proficiency and to explore the impact of the training on the frequency of participants’ engagement in online activities (eg, shopping, banking, and accessing government services) and on their confidence, as well as to assess their satisfaction with, and acceptability of, the program. Overall, participants’ mobile device proficiency was higher after the program than at baseline, and they were going online more frequently for some activities; however, confidence was unchanged. Program acceptability was high, and participants who started or completed some lessons as well as those who were still accessing the Gluu Essentials resources had the greatest changes in proficiency.

Participants in the Gluu Essentials digital skills training program were middle-aged and older adults interested in learning about tablets; indeed, the Gluu Essentials program is targeted at basic tablet skills specifically, although there are a few broader digital skills touched on, such as digital security as well as Facebook and Zoom basics. As such, we found positive changes in mobile device proficiency after the program. Overall mobile device proficiency reflected increases primarily in the subscale competencies of mobile device basics, communication, internet, and troubleshooting. The increase in troubleshooting scores is noteworthy because it reflects participants’ growing capacity to problem solve issues that arise without dependence on others, such as family and friends. In a recent scoping review, the lack of technical support that included troubleshooting was highlighted as a barrier to using technology for older adults [14]. Gluu Essentials program participants evidently received the support they needed from the program to demonstrate an improvement in their ability to troubleshoot.

In addition to increased mobile device proficiency, we found that there were increases in the frequency of going online for shopping and accessing government services among the participants. It is possible that having achieved greater mastery of their devices combined with the fact that mobile devices are *handy*, participants increased the frequency with which they went online for these activities. By contrast, going online for COVID-19–related information was reduced after the training, which was likely a reflection of the timing of most of the follow-up surveys because COVID-19–related measures lessened in Canada in the summer of 2022, although the COVID-19 pandemic was not officially downgraded until May 4, 2023 [40]. Similar to other research [41,42], we found that going online for email was so ubiquitous that there was little room for improvement. Our study also reported that going online for information was already fairly common at baseline. Going online for banking increased, although not significantly, and going online for emergency services did not change. Interventions targeted more directly at how to engage with emergency preparedness services may be needed to increase this specific activity.

Likewise, confidence in going online and avoiding fraud and scams was not significantly higher at follow-up than at baseline. It could be that training targeted more to these specific skills (especially for confidence in avoiding frauds and scams) is needed to improve confidence in these areas because digital security was only a small component of the Gluu Essentials program. Indeed, the Gluu Essentials digital skills training program and manual were intended to build proficiency—the essentials of actually using a tablet. Nevertheless, it is perplexing that confidence in going online did not increase, especially because the participants were going online more frequently for some activities. Other similar digital skills training programs for older adults improved self-efficacy [43]. Research indicates that repetition and opportunities to practice along with ongoing support are needed to build confidence [18,20,44]. In a recent cross-sectional study, social influence (ie, opinions of others about using the internet) was a bigger predictor of self-efficacy than social support (ie, having someone to help) [45], and social influence may play a role in motivating older adults to seek training. Indeed, reflected in our participants’ open-ended responses was a desire for greater support. In a qualitative study of 10 older adults, confidence was bolstered by positive experiences (eg, successfully figuring out how to use an app) and opportunities to integrate personal interests (eg, photos, art, and games) in weekly technology training sessions, whereas frustrating experiences (eg, being unable to solve a problem) eroded confidence [46]. In future, adding opportunities in the Gluu Essentials digital skills training program for participants to engage with personal interests and support to build positive experiences might build greater confidence. Despite several studies conducted to improve mobile device proficiency among older adults, several others describe the lack of technology engagement among this population. Indeed, among older adults in Canada, age is the primary determinant of internet use, with only 40% of seniors aged >80 years using the internet in 2016 [47]. Tailored rather than one-size-fits-all programming may be needed for different age cohorts of older adults. In the study by Roque and Boot [9], younger adults scored nearly twice as high as older adults on all MDPQ subscales; our sample was more advanced in terms of mobile device proficiency than the older adult sample in the study by Roque and Boot [9] (mean scores ranged from 2.3 to 3.4) but not as proficient as their young adult sample (mean scores ranged from 4.7 to 4.9).

Of the 143 participants who responded, 117 (81.8%) made use of the printed workbooks (monthly, weekly, and daily) that accompanied the training, and 78% (74/95) believed that the workbook was an important component of their learning; in addition, qualitative feedback from the workbook users indicated their need for it and its helpfulness. This aligns with qualitative evidence that older adults prefer using self-training text materials, such as a manual [48]. However, many of the participants (53/143, 37.1%) used the workbook once a month or less, which may have reflected the less structured lesson delivery for online participants who were mailed printed workbooks. Finally, only a few participants (29/138, 21%) accessed digital coaches as part of the program, and the participants’ open-ended responses identified their need for more real-life support. Indeed, 48 (33.1%) of the 145 follow-up survey participants had completed paper surveys at baseline, meaning they were engaged in person through a community organization, yet only 21 (44%) of these 48 participants reported having a digital coach or digital classes. It is possible that some of the participants may have misunderstood the digital coach question and not interpreted their local organization’s support as being the equivalent of the role of a digital coach. It is also possible that some organizations simply handed out the workbooks and surveys but did not recruit volunteers to complete the Gluu Essentials training and act as digital coaches. In the future, a mechanism to ensure that community organizations are prepared to support learners may improve program fidelity.

Although the majority of participants (101/144, 70.1%) reported increased mobile device proficiency at follow-up compared with baseline, notably, several participants (43/144, 29.9%) reported a slight decrease in proficiency. It is possible that some participants did not have the one-to-one support that they were asking for or felt overwhelmed with the unpaced volume of information and how they could practice as was noted in some open-text responses. Furthermore, engagement with many of the program components was not related to mobile device proficiency, and this may reflect the smaller number of participants who reported engagement (eg, with a digital coach), or it could reflect the variability among learners (eg, those struggling the most needing to refer to the workbook more frequently than the others); however, encouragingly, those who engaged with the 12 Gluu Essentials lessons did report a greater increase in mobile device proficiency than those who did not start any lessons, reflecting the impact of the workbook. Indeed, engaging with the content or lessons might be considered foundational to all other program resources; other program supports (such as coaches) cannot provide optimal support if learners have not engaged with the lessons. Furthermore, continuing to access the Gluu Essentials resources was related to increased mobile device proficiency; therefore, ongoing engagement and practice are likely important for success.

Despite variation in self-reported program engagement, the majority of the participants did find the program acceptable, with more than two-thirds reporting being satisfied (82/93, 88%), having learned new things (75/92, 82%), and being more willing to use technology for their aging plan (60/87, 69%). Likewise, 79% (76/96) rated the usability of the Gluu Essentials programming as *above average*. Continued research is needed on middle-aged and older adults’ attitudes toward, and satisfaction with, digital skills training, specifically with individual training components, to inform the development of relevant and acceptable programming [16,48].

This study focused primarily on what has been referred to as the *second-level* digital divide, namely having the skills and literacy to access information and communication technology [49]; however, the open-ended feedback from participants also highlighted cost—of devices as well as the internet—as significant barriers to access, suggesting that, at least for some, the first-level divide in access is still a challenge. Future work is needed that offers digital literacy training alongside access to devices and connectivity at low or no cost to begin to reduce these inequities.

### Strengths and Limitations

This study has several strengths. The Gluu Essentials digital skills training program was implemented in the *real world*, that is, community organizations and participants adopted the training, and the program was delivered voluntarily. This allowed for an assessment of the potential and effectiveness of digital skills training in a natural environment, which may be influenced by external variables (eg, available support and resources, motivation, location or transportation, and cost) that are not necessarily apparent in highly controlled or specific settings. Furthermore, the assessment of program engagement allowed us to examine the impacts of different program components on change in mobile device proficiency, illuminating the importance of engagement with the core programming (ie, lessons).

Despite these strengths, this study has several limitations. Participants were motivated to learn digital skills, and although training was free, participants had to have their own device or have a means of obtaining a device (eg, through senior service organizations or family or friends); therefore, the results may not generalize to other populations. In addition, as this was an ongoing and freely offered community organization–delivered program, we were not able to use a randomized controlled study design. Relatedly, owing to challenges with the organizations tracking the distribution of the paper surveys, we were unable to report the baseline survey response rate as well as the total number of participants engaged by the community organizations during the study period. Likewise, our inability to document how and in what structure the organizations offered the training was another limitation. Despite efforts to mail follow-up paper surveys to participants who had completed paper surveys at baseline, more participants who had completed paper surveys at baseline were lost to follow-up overall. However, those lost to (survey) follow-up did not necessarily drop out of training; all paper baseline survey completers received a printed workbook and had some level of support from their community organizations. Engaging community organizations to distribute paper follow-up surveys may have improved response rates. Follow-up survey completers also had higher mobile device proficiency scores at baseline; despite this, our follow-up participants represented the full range of mobile device proficiency scores (ranging from 1 to 5). Likewise, we retained some participants who *never* went online for each activity at baseline (just proportionately fewer of them). We also observed increases in mobile device proficiency and the frequency of going online for 2 activities, and linear mixed model analyses suggested that the observed differences could have been even greater if more participants with lower scores at baseline had been retained at follow-up.

### Conclusions

This study is unique in that we examined the impact of an existing digital literacy training program on middle-aged and older adults’ digital engagement. Middle-aged and older adults participating in a real-life implementation of the program had higher follow-up scores on mobile device proficiency and greater frequency of going online for shopping and accessing government services compared with the baseline. The Gluu Essentials program was an acceptable and effective approach to digital skills acquisition, and future efforts should focus on strengthening program delivery to ensure that all components are available to all participants. Altogether, these findings advance the understanding of middle-aged and older adults’ digital skills acquisition, particularly with respect to learning to use mobile devices, and have implications for other programs designed to engage and support middle-aged and older adults.

## Appendix

Multimedia Appendix 1 Characteristics at baseline of all participants, completers, and participants missing from follow-up.

Multimedia Appendix 2 Proportion of participants (n=145) who completed both baseline and follow-up surveys and reported engaging in online activities at any frequency above never.

## Abbreviations

MDPQ: Mobile Device Proficiency Questionnaire
