# Supplementary material for: Gluu Essentials Digital Skills Training for Middle-Aged and Older Adults That Makes Skills Stick: Results of a Pre-Post Intervention Study
Source: JMIR Aging. 2023 Nov 10;6:e50345. doi: 10.2196/50345 (PMC10674153; doi:10.2196/50345)
Supplement: Multimedia Appendix 1 [file aging_v6i1e50345_app1.docx]

**Supplementary Appendix 1**

Characteristics at baseline of all participants, completers, and participants missing from follow-up

|  | **All Participants at Baseline**  (n=270) | **Follow-up Completers**  (n=145) | **Participants Missing Follow-up**  (n=125) | *p*^a^ |
| --- | --- | --- | --- | --- |
|  | M(SD) | M(SD) | M(SD) |  |
|  |  |  |  |  |
| **MDPQ – Total Scale Score** | 3.77 (1.00) | 3.93 (0.91) | 3.58 (1.08) | .005 |
| **MDPQ Subscales:** |  |  |  |  |
| Mobile Device Basics | 4.16 (1.01) | 4.32 (0.87) | 3.98 (1.13) | .007 |
| Communication | 4.21 (1.05) | 4.32 (0.94) | 4.08 (1.16) | .066 |
| Internet | 3.94 (1.13) | 4.13 (0.99) | 3.72 (1.25) | .005 |
| Calendar | 3.43 (1.51) | 3.58 (1.48) | 3.25 (1.54) | .080 |
| Entertainment | 3.33 (1.38) | 3.49 (1.33) | 3.15 (1.42) | .045 |
| Privacy | 3.31 (1.19) | 3.55 (1.10) | 3.03 (1.24) | < .001 |
| Troubleshooting | 3.74 (1.14) | 3.91 (1.08) | 3.53 (1.18) | .007 |
| **Confidence going online** | 3.61 (0.96) | 3.74 (0.92) | 3.46 (0.99) | .019 |
| **Confidence frauds/scams** | 2.89 (0.91) | 3.04 (0.73) | 2.72 (1.07) | .006 |
|  | n(%) | n(%) | n(%) | *p*^b^ |
|  |  |  |  |  |
| **Email frequency** |  |  |  | < .001 |
| Never | 13 (4.8%) | 2 (1.4%) | 11 (8.8%) |  |
| Once a year | 0 (0.0%) | 0 (0.0%) | 0 (0.0%) |  |
| Several times a year | 4 (1.5%) | 1 (0.7%) | 3 (2.4%) |  |
| Once a month | 6 (2.2%) | 3 (2.1%) | 3 (2.4%) |  |
| Several times a month | 8 (3.0%) | 1 (0.7%) | 7 (5.6%) |  |
| Once a week | 4 (1.5%) | 2 (1.4%) | 2 (1,6%) |  |
| Several times a week | 38 (14.1%) | 16 (11.0%) | 22 (17.6%) |  |
| Once a day | 42 (15.6%) | 27 (18.6%) | 15 (12.0%) |  |
| Several times a day | 149 (55.2%) | 92 (63.4%) | 57 (45.6%) |  |
| Missing | 6 (2.2%) | 1 (0.7%) | 5 (4.0%) |  |
| **Online shopping frequency** |  |  |  | .150 |
| Never | 54 (20.0%) | 25 (17.2%) | 29 (23.2%) |  |
| Once a year | 22 (8.1%) | 10 (6.9%) | 12 (9.6%) |  |
| Several times a year | 68 (25.2%) | 40 (27.6%) | 28 (22.4%) |  |
| Once a month | 30 (11.1%) | 18 (12.4%) | 12 (9.6%) |  |
| Several times a month | 42 (15.6%) | 25 (17.2%) | 17 (13.6%) |  |
| Once a week | 13 (4.8%) | 9 (6.2%) | 4 (3.2%) |  |
| Several times a week | 16 (5.9%) | 8 (5.5%) | 8 (6.4%) |  |
| Once a day | 4 (1.5%) | 2 (1.4%) | 2 (1.6%) |  |
| Several times a day | 0 (0.0%) | 0 (0.0%) | 0 (0.0%) |  |
| Missing | 21 (7.8%) | 8 (5.5%) | 13 (10.4%) |  |
| **Online banking frequency** |  |  |  | .170 |
| Never | 59 (21.9%) | 24 (16.6%) | 35 (28.0%) |  |
| Once a year | 0 (0%) | 0 (0.0%) | 0 (0.0%) |  |
| Several times a year | 8 (3.0%) | 5 (3.4%) | 3 (2.4%) |  |
| Once a month | 23 (8.5%) | 12 (8.3%) | 11 (8.8%) |  |
| Several times a month | 53 (19.6%) | 32 (22.1%) | 21 (16.8%) |  |
| Once a week | 36 (13.3%) | 23 (15.9%) | 13 (10.4%) |  |
| Several times a week | 59 (21.9%) | 33 (22.8%) | 26 (20.8%) |  |
| Once a day | 12 (4.4%) | 8 (5.5%) | 4 (3.2%) |  |
| Several times a day | 8 (3.0%) | 2 (1.4%) | 6 (4.8%) |  |
| Missing | 12 (4.4%) | 6 (4.1%) | 6 (4.8%) |  |
| **Access Gov’t services** |  |  |  | < .001 |
| Never | 71 (26.7%) | 25 (17.2%) | 47 (37.6%) |  |
| Once a year | 30 (11.1%) | 15 (10.3%) | 15 (12.0%) |  |
| Several times a year | 103 (38.1%) | 65 (44.8%) | 38 (30.4%) |  |
| Once a month | 30 (11.1%) | 18 (12.4%) | 12 (9.6%) |  |
| Several times a month | 23 (8.5%) | 17 (11.7%) | 6 (4.8%) |  |
| Once a week | 6 (2.2%) | 3 (2.1%) | 3 (2.4%) |  |
| Several times a week | 1 (0.4%) | 1 (0.7%) | 0 (0.0%) |  |
| Once a day | 0 (0.0%) | 0 (0.0%) | 0 (0.0%) |  |
| Several times a day | 0 (0.0%) | 0 (0.0%) | 0 (0.0%) |  |
| Missing | 5 (1.9%) | 1 (0.7%) | 4 (3.2%) |  |
| **Search for information online** |  |  |  | .011 |
| Never | 28 (10.4%) | 8 (5.5%) | 20 (16.0%) |  |
| Once a year | 7 (2.6%) | 2 (1.4%) | 5 (4.0%) |  |
| Several times a year | 35 (13.0%) | 22 (15.2%) | 13 (10.4%) |  |
| Once a month | 27 (10.0%) | 16 (11.0%) | 11 (8.8%) |  |
| Several times a month | 69 (25.6%) | 36 (24.8%) | 33 (26.4%) |  |
| Once a week | 27 (10.0%) | 14 (9.7%) | 13 (10.4%) |  |
| Several times a week | 56 (20.7%) | 35 (24.1%) | 21 (16.8%) |  |
| Once a day | 5 (1.9%) | 4 (2.8%) | 1 (0.8%) |  |
| Several times a day | 9 (3.3%) | 7 (4.8%) | 2 (1.8%) |  |
| Missing | 7 (2.6%) | 1 (0.7%) | 6 (4.8%) |  |
| **Access COVID- information** |  |  |  | .001 |
| Never | 41 (15.2%) | 13 (9.0%) | 28 (22.4%) |  |
| Once a year | 16 (5.9%) | 7 (4.8%) | 9 (7.2%) |  |
| Several times a year | 117 (43.3%) | 72 (49.7%) | 45 (36.0%) |  |
| Once a month | 32 (11.9%) | 19 (13.1%) | 13 (10.4%) |  |
| Several times a month | 25 (9.3%) | 19 (13.1%) | 6 (4.8%) |  |
| Once a week | 8 (3.0%) | 5 (3.4%) | 3 (2.4%) |  |
| Several times a week | 5 (1.9%) | 2 (1.4%) | 3 (2.4%) |  |
| Once a day | 1 (0.4%) | 1 (0.7%) | 0 (0.0%) |  |
| Several times a day | 0 (0.0%) | 0 (0.0%) | 0 (0.0%) |  |
| Missing | 25 (9.3%) | 7 (4.8%) | 18 (14.4%) |  |
| **Access emergency/prepared** |  |  |  | .794 |
| Never | 81 (30.0%) | 42 (29.0%) | 39 (31.2%) |  |
| Once a year | 29 (10.7%) | 18 (12.4%) | 11 (8.8%) |  |
| Several times a year | 81 (30.0%) | 46 (31.7%) | 35 (28.0%) |  |
| Once a month | 28 (10.4%) | 15 (10.3%) | 13 (10.4%) |  |
| Several times a month | 20 (7.4%) | 13 (9.0%) | 7 (5.6%) |  |
| Once a week | 2 (0.7%) | 1 (0.7%) | 1 (0.8%) |  |
| Several times a week | 5 (1.9%) | 0 (0.0%) | 5 (4.0%) |  |
| Once a day | 2 (0.7%) | 2 (1.4%) | 0 (0.0%) |  |
| Several times a day | 0 (0.0%) | 0 (0.0%) | 0 (0.0%) |  |
| Missing | 22 (8.1%) | 8 (5.5%) | 14 (11.2%) |  |

Note: ^a^p value based on Independent t-tests comparing follow-up completers versus non-completers; ^b^p value based on Mann-Whitney U tests comparing follow-up completers versus non-completers on baseline frequency of going online variables (with missing data excluded).
