# Supplementary figures and images for: Gluu Essentials Digital Skills Training for Middle-Aged and Older Adults That Makes Skills Stick: Results of a Pre-Post Intervention Study
Source: JMIR Aging. 2023 Nov 10;6:e50345. doi: 10.2196/50345 (PMC10674153; doi:10.2196/50345)

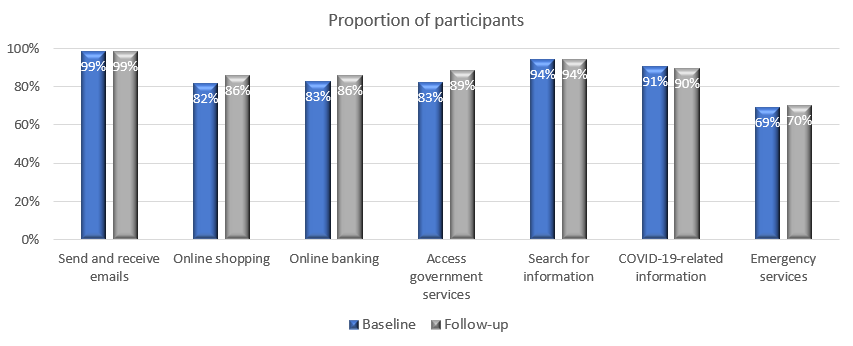

Supplement: Multimedia Appendix 2 [file aging_v6i1e50345_app2.png]
